# Unraveling Differential Transcriptomes and Cell Types in Zebrafish Larvae Intestine and Liver

**DOI:** 10.3390/cells11203290

**Published:** 2022-10-19

**Authors:** Yuqi Gao, Qingxia Jin, Ce Gao, Yayue Chen, Zhaoxiang Sun, Guoji Guo, Jinrong Peng

**Affiliations:** 1 MOE Key Laboratory for Molecular Animal Nutrition, College of Animal Sciences, Zhejiang University, Hangzhou 310058, China; 2 College of Life Sciences, Zhejiang University, Hangzhou 310058, China; 3 School of Medicine, Zhejiang University, Hangzhou 310058, China

**Keywords:** enterocytes, hepatocytes, intestine development, liver development, RNA-seq, scRNA-seq, transcription factors, zebrafish

## Abstract

The zebrafish intestine and liver, as in other vertebrates, are derived from the endoderm. Great effort has been devoted to deciphering the molecular mechanisms controlling the specification and development of the zebrafish intestine and liver; however, genome-wide comparison of the transcriptomes between these two organs at the larval stage remains unexplored. There is a lack of extensive identification of feature genes marking specific cell types in the zebrafish intestine and liver at 5 days post-fertilization, when the larval fish starts food intake. In this report, through RNA sequencing and single-cell RNA sequencing of intestines and livers separately dissected from wild-type zebrafish larvae at 5 days post-fertilization, together with the experimental validation of 47 genes through RNA whole-mount in situ hybridization, we identified not only distinctive transcriptomes for the larval intestine and liver, but also a considerable number of feature genes for marking the intestinal bulb, mid-intestine and hindgut, and for marking hepatocytes and cholangiocytes. Meanwhile, we identified 135 intestine- and 97 liver-enriched transcription factor genes in zebrafish larvae at 5 days post-fertilization. Our findings provide rich molecular and cellular resources for studying cell patterning and specification during the early development of the zebrafish intestine and liver.

## 1. Introduction

The digestive tract and accessory organs (liver and pancreas) form the digestive system, which is specified and developed from distinct regions of the endoderm (e.g., in mice, the stomach, liver and pancreas originate from the foregut endoderm, the small intestine originates from the midgut endoderm and the large intestine originates from the hindgut endoderm) [1,2,3]. In zebrafish, endoderm cells emerge first as individuals mingled with mesoderm cells. Later, the endoderm cells migrate to form funnel-shaped cell aggregates along the midline at ~24 h post-fertilization (hpf). The lower part of the funnel-shaped endoderm develops further to yield a discernable intestinal tube, liver bud and pancreatic bud at ~50 hpf [4,5,6,7,8,9,10,11,12,13,14]. By 5 days post-fertilization (dpf), with the mouth opening, the zebrafish digestive system has been well established, marked by a fully functional liver and a clearly patterned intestinal tube in the order of, from anterior to posterior, the intestinal bulb, mid-intestine, hindgut and proctodeum, four functional domains [3,5,8,14]. The development and functional specification of the zebrafish digestive system is orchestrated by multiple genetic networks formed by signaling molecules and transcription factors (TFs) [1,2,10,14,15,16]. It is worth mentioning that the zebrafish is stomachless and the intestinal bulb is thought to be an analog of the stomach, while the hepatopancreatic duct is fused to the rostral region of the intestinal bulb [8,14,17,18].

Previous studies have shown that intestinal cells from the intestinal bulb toward the proctodeum express regionalized specific genes to execute different physiological functions, such as food digestion and nutrient absorption [3,19,20,21,22]. For example, Wang and colleagues separated the intestine of an adult zebrafish into seven segments and followed the microarray approach to compare the gene expression patterns. They found that sections 1 to 5 (anatomically covering the intestinal bulb and mid-intestine) shared similar transcriptomes, while sections 6 and 7 (mainly the hindgut together with the proctodeum) were clustered into one group [20]. Lickwar and colleagues compared the genome-wide mRNA and accessible chromatin data from the intestines of adult zebrafish, sticklebacks, mice and humans. They found that there was a remarkable conservation of genes expressed along the intestine among these four species and identified many differentially accessible and cross-species-conserved regulatory regions in the intestine [3]. They divided the zebrafish intestine, from the anterior toward the proctodeum, into five sub-regions, including *ada^+^*, *fabp2^+^ rbp2a^+^*, *fabp6^+^ slc10a2^+^*, *lamp2^+^* and caudal intestine [3]. Recently, for the purpose of studying the microbe response processes in the zebrafish intestine, Willms and colleagues pooled the intestine, liver and pancreas together from 6 dpf-old zebrafish larvae for single-cell RNA sequencing (scRNA-seq) analysis. They annotated 18 cell types from the 35 cell clusters identified by scRNA-seq, including hepatocytes, acinar cells, enterocytes, goblet cells, endocrine cells, tuft cells, etc. [21]. Although complicated by the cells from the liver and pancreas, their detailed analysis showed that enterocytes, in addition to the lysosome-rich enterocytes (LRE) [19], were composed of five cell clusters. Unfortunately, they did not use feature genes to perform in situ hybridization to locate these cells within the intestine [21]. Despite these valuable works, knowledge of the gene expression profiles, cell types and their molecular features in the zebrafish larvae intestine and liver at 5 dpf is yet to be fully elucidated, mainly due to the difficulty and tedious labor of separating the tiny tissues at the embryonic stage. Deciphering the transcriptomes and cell identities in the larval intestine and liver separately will no doubt provide new molecular and cellular signatures for studying the specification and developmental regulation of the digestive system.

In this report, we micro-dissected the intestinal tube and liver buds, respectively, from zebrafish larvae at 5 dpf and then obtained satisfactory RNA sequencing (RNA-seq) data from these samples. The RNA-seq data allowed us to identify not only the organ-specific functional genes, but also the corresponding spectrum of TF genes expressed in the intestine and liver at 5 dpf. We also obtained the RNA-seq data for three segments of the intestine (anterior, middle and posterior) at 5 dpf, which allowed us to acquire a more elaborate molecular view of the functional specialization of these regions at 5 dpf. Next, we acquired the scRNA-seq data from the micro-dissected intestine and liver at 5 dpf and annotated 10 cell types in the intestine and 8 cell types in the liver. For the intestine, 3 of the 10 cell types represented the regionalized enterocytes along the intestine at 5 dpf. In addition, mapping the 385 intestine-specific genes identified by our RNA-seq analysis (intestine vs. liver: fold-change > 4 and transcripts per million (TPM) > 50) to the 10 intestine cell types by a feature plot unraveled their cellular specificity. In total, the expression patterns of 47 genes were validated by whole-mount in situ hybridization (WISH). These findings not only extend our understanding of the molecular and cellular features in the larval intestine and liver, but also provide a rich resource for studying the process of organogenesis and development of the intestine and liver.

## 2. Materials and Methods

### 2.1. Fish Lines and Maintenance, Ethics Statement

The AB wild-type (WT) zebrafish strain was used in this study. Fertilized eggs were placed in a petri dish containing egg water and allowed to grow to 5 dpf at 28 °C. All animal procedures were adopted in full accordance with the requirement by the ‘Regulation for the Use of Experimental Animals in Zhejiang Province’. This work was approved by the Animal Ethics Committee in the School of Medicine, Zhejiang University (ETHICS CODE Permit NO. ZJU2011-1-11-009Y). The *pparda^zju1^* mutant was generated using the CRISPR-Cas9 method with gRNA (5′-GAACGGGCTGGTCTGGAACC-3′) specifically targeting *pparda*.

### 2.2. WISH

Embryos were fixed in 4% PFA (PBS) for 12 h at 4 °C. WISH probes were labeled with digoxigenin (DIG, Roche Diagnostics, Basel, Switzerland) according to the manufacturer’s instructions. WISH was performed as previously described [23]. To generate the WISH probes, PCR primers were designed based on available gene sequence data (Table S1) and PCR products were purified and confirmed by sequencing prior to the generation of the WISH probe. Images of the WISH embryos were captured under a Nikon microscope (AZ100, Nikon, Tokyo, Japan).

### 2.3. RNA-Seq and Data Analysis

The intestine tissue or liver bud were dissected from 5 dpf-old zebrafish embryos. Intestine tissues from ~30 embryos or liver buds from ~50 embryos were pooled and then treated with TRIZOL for total RNA extraction. The micro-dissected intestine was also cut into three fragments corresponding to the anterior, middle and posterior portions of the intestine, and the fragments were pooled for total RNA extraction. The obtained total RNA (~200–400 ng/sample) was used for library construction via the Smart-seq2 protocol as described in [24]. Library sequencing was performed on an Illumina HiSeq X Ten PE150 platform (Illumina, San Diego, CA, USA). RNA sequencing reads were aligned with HISAT2 (version 2.1.0) to the zebrafish genome (GRCz11) and ENSEMBL v99 annotations [25,26,27].

Analysis of the differentially expressed genes (DEGs) was conducted with DESeq2 (v1.28.1) and gene ontology (GO) set overrepresentation analysis was performed with ClusterProfiler (v3.16.1) [28,29]. TFBS analysis for the identification of TF regulators was performed by David Bioinformatics Resources [30].

### 2.4. scRNA-Seq and Data Analysis

The scRNA-seq of the intestine tissue and liver bud was performed following the microwell-Seq approach in this study [31]. For each sample, intestine tissues or liver buds dissected from ~50 embryos at 5 dpf were pooled and digested with ACCUTASE (Yeasen Biotechnology, Shanghai, China). A single-cell library was generated as described [31,32]. Library sequencing was performed on an Illumina HiSeq X Ten PE150 platform. Aligned reads and gene barcode matrices were then generated from FASTQ files, including Read 1 and Read 2, using dropEst (v0.8.5) (https://dropest.readthedocs.io/en/v0.8.5/dropest.html). Further analyses were performed with the R package Seurat (v4.0, Vienna, Austria) (https://mran.microsoft.com/snapshot/2020-05-30/bin/windows/base/). A threshold of unique counts over 2500 or less than 200 was set to filter cell doublets. Low-quality cells that had >20% mitochondrial counts were filtered.

## 3. Results

### 3.1. Identification of DEGs between the Intestine and Liver at 5 dpf

**Figure 1:**
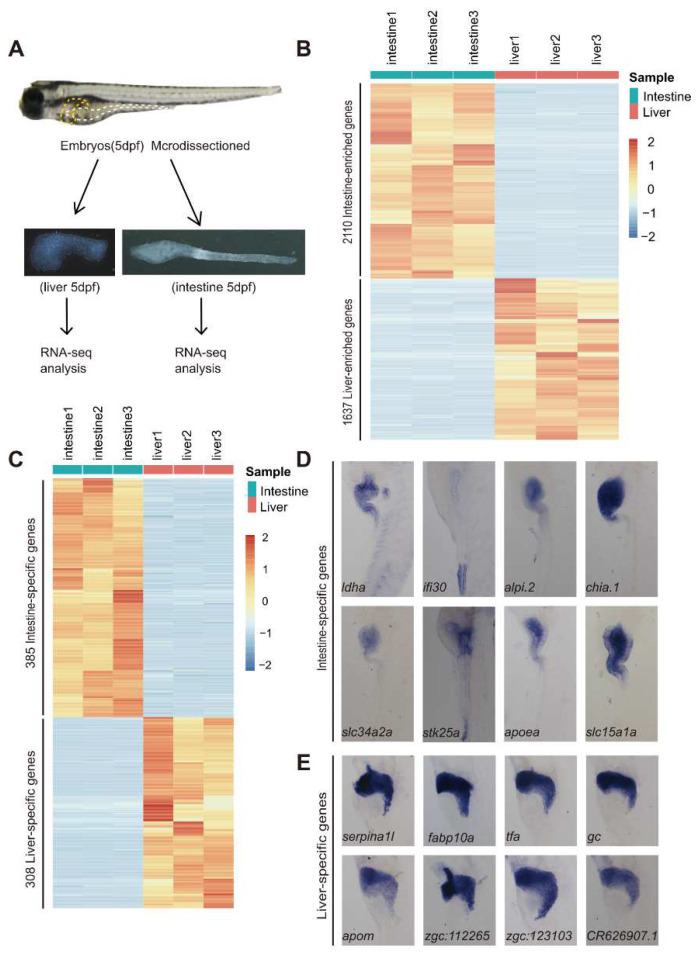
Identification of DEGs between the 5 dpf larval intestine and liver by RNA-seq analysis. (**A**) Flow chart showing the micro-dissection of the intestinal tube and liver bud from the zebrafish embryos at 5 dpf. The intestinal tube and liver bud are outlined with white and yellow dashed lines, respectively, in the embryo. (**B**) Heatmap showing the hierarchical clustering of the 2110 intestine-enriched and 1637 liver-enriched DEGs in the three intestine and three liver RNA-seq samples. (**C**) Heatmap showing the hierarchical clustering of the 385 intestine-specific and 308 liver-specific genes. (**D**,**E**) WISH showing the expression of 8 intestine-specific genes (**D**) and 8 liver-specific genes (**E**). Note that the *ifi30* transcripts were enriched in the hindgut. The abbreviations of gene names followed the ENSEMBL naming system.

We micro-dissected the intestine (from the intestinal bulb to the proctodeum) from the WT zebrafish embryos at 5 dpf and obtained three independent samples by pooling intestine tissue from ~30 embryos in each sample (Figure 1A). Similarly, three independent liver samples, each by pooling micro-dissected liver buds from ~50 embryos at 5 dpf, were obtained (Figure 1A). The total RNA was extracted from these samples and subjected to RNA-seq analysis. The clean bases in the three intestine and three liver samples all exceeded 12 GB. Data filtering using the Clean Q30 Bases Rate program revealed that the Q30 for these six samples was between 86.5% and 93.1% (Table S2). Aligning the clean reads to the zebrafish genome (GRCz11) (http://ftp.ensembl.org/pub/release-107/, accessed on 14 May 2022) showed that 58.4–63.4% and 80–81.9% of the total clean reads from the three intestine and three liver samples, respectively, were mapped to the zebrafish genome (Table S2). 

The expression level of each gene in each sample was calculated based on the TPM. In total, 19,653, 18,853 and 20,820 genes in the three intestine samples and 21,378, 21,774 and 21,802 genes in the three liver samples (rowSums (counts) > 1) were identified (Table S2; National Genomics Data Centre accession number: PRJCA006909). Unsupervised clustering showed that the three intestine samples were clustered as one clad, while the three liver samples were clustered as another (Figure S1). Through comparing the RNA-seq data between the WT intestine and liver (|log_2_ (fold-change)| > 2 and *p* < 0.01 in intestine or liver), we found that a total of 2110 genes were more abundant in the intestine (termed as intestine-enriched), while 1637 genes were enriched in the liver (termed as liver-enriched) (Figure 1B; Tables S3 and S4).

To identify a spectrum of marker genes more specifically defining the intestine and liver at 5 dpf, we applied more stringent criteria (TPM > 50 in intestine or liver) to the 2110 intestine- and 1637 liver-enriched genes. In total, 385 intestine-enriched and 308 liver-enriched genes passed the criteria and were arbitrarily classified as the embryonic intestine-specific and liver-specific genes, respectively (Figure 1C; Tables S5 and S6). The majority of these genes are well-known genes specifically functioning in the intestine (e.g., *cdx1b*, *fabp2*, *fabp6,* etc.) or liver (e.g., *apoa2*, *bhmt*, *fabp10a,* etc.) [3,20,33], whereas many others are yet to be characterized as the intestine- or liver-specific genes by checking their records in Zebrafish Information Network (ZFIN) (http://zfin.org) (e.g., for the intestine, *slc15a1a*, *dpep1*, *cuzd1.1*, *si:ch211-202h22.9* and *si:ch211-133l5.7*; for the liver, *wu:fj16a03*, *si:ch211-284e20.8*, *miox*, *mlsl* and *si:ch211-186e20.7*). WISH confirmed the specific expression of the well-known intestinal genes *ldha*, *ifi30*, *alpi.2*, *chia.1*, *slc34a2a* and *stk25* (Figure 1D) and the liver genes *serpina1l*, *fabp10a*, *tfa*, *gc*, *apom*, *zgc112265* and *zgc123103* at 5 dpf (Figure 1E). Meanwhile, WISH verified *slc15a1a* to be novel intestine-specific (Figure 1D) and *CR626907.1* to be liver-specific genes (Figure 1E).

### 3.2. Unraveling Differential Functional Processes in the Intestine and Liver at 5 dpf by GO Analysis 

**Figure 2:**
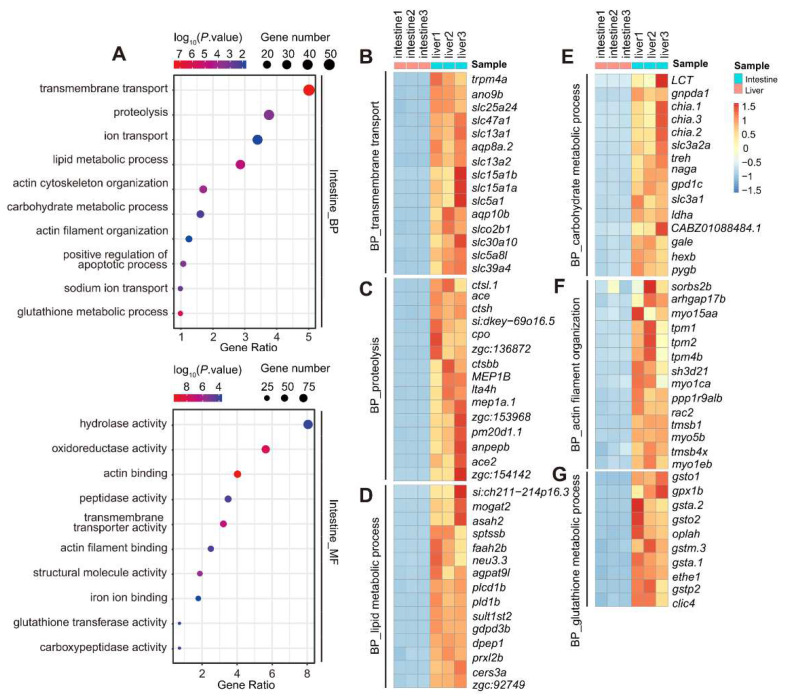
GO analysis of the 2110 intestine-enriched DEGs identified at 5 dpf. (**A**) GO analysis of the 2110 intestine-enriched DEGs under the GO_BP (upper panel) and GO_MF (lower panel) terms. Top 10 categories are shown here. (**B**–**G**) Heatmap showing the hierarchical clustering of representative DEGs in the categories (chosen from the top 10 under the GO_BP term) of ‘transmembrane transport’ (**B**), ‘proteolysis’ (**C**), ‘lipid metabolic process’ (**D**), ‘carbohydrate metabolic process’ €, ‘actin cytoskeleton/filament organization’ (**F**) and ‘glutathione metabolic pathway’ (**G**) in the three intestine and three liver RNA-seq samples.

GO analysis of the 2110 intestine-enriched genes based on the biological process (GO_BP) and molecular function (GO_MF) terms resulted in, though in different orders, similar top 10 categories (Figure 2A). We analyzed the top 10 categories in the GO_BP term in detail. The first category was ‘transmembrane transport’, containing 56 genes that mainly encoded different solute carriers, such as *slc5a1*, *slc15a1b*, *slc18b1*, *slc25a24*, etc. (Figure 2B; Table S7). Others included ‘proteolysis’ (42 genes, encoding various peptidases, such as *asrgl1*, *capn2b*, *cndp2*, *cpo*, etc.) (Figure 2C; Table S7), ‘lipid metabolic process’ (32 genes, such as *acot19*, *gdpd3b*, *mogat2*, *plcd1b*, *pld1b*, etc.) (Figure 2D; Table S7) and ‘carbohydrate metabolic process’ (18 genes, including *gale*, *treh*, *pgm3*, *pygb*, *chia.1*, etc.) (Figure 2E; Table S7). Interestingly, two categories, ‘actin cytoskeleton organization’ (19 genes, for actin filament assembly, including *flna*, *smtna*, *pfn1*, *actn1*, *pdlim1*, *pdlim7*, etc.) and ‘actin filament organization’ (14 genes, mainly encoding different myosins and their regulators, such as *tpm2*, *myo15aa*, *myo5b*, *myo1ca*, *myo1eb*, etc.) were also among the top 10 categories (Figure 2F; Table S7), reflecting an active smooth muscle system controlling the contraction of the digestive tract in zebrafish. In addition, the identification of the ‘glutathione metabolic pathway’ (11 genes, including *gsta.1*, *gsta.2*, *gsto1*, *gsto2*, *gstp2*, etc.) among the top 10 categories suggested that the intestine exhibited high antioxidant activities (Figure 2G; Table S7). The enrichment of these pathways is fully consistent with the known function of the intestine for food digestion, nutrient absorption and innate immunity [3,19,20].

**Figure 3:**
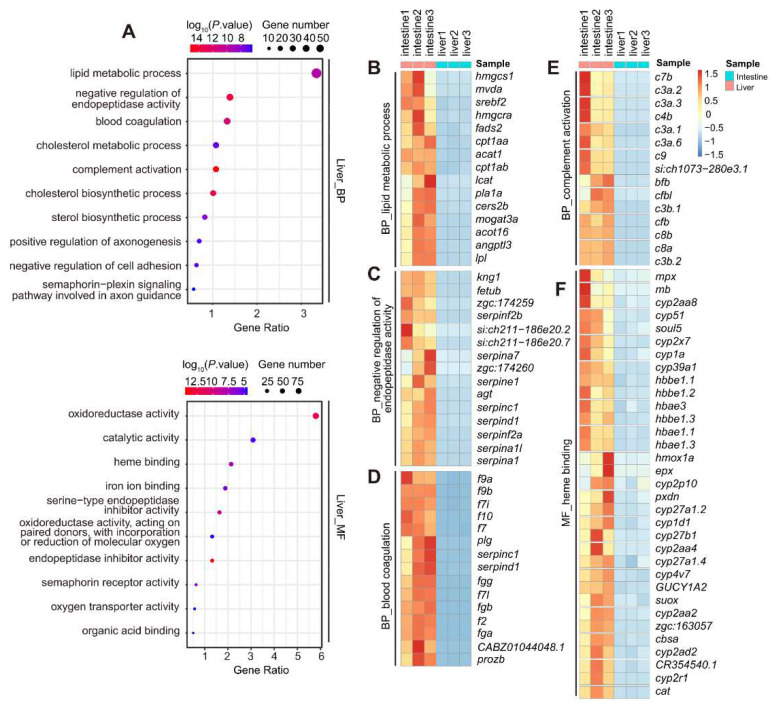
GO analysis of the 1637 liver-enriched DEGs identified at 5dpf. (**A**) GO analysis of the 1637 liver-enriched DEGs under the GO_BP (upper panel) and GO_MF (lower panel) terms. Top 10 categories are shown here. (**B**–**F**) Hierarchical clustering heatmap comparing representative genes in the categories of ‘lipid metabolic process’ (**B**), ‘negative regulation of endopeptidase activity’ (**C**), ‘blood coagulation’ (**D**) and ‘complement activity’ (**E**) under the GO_BP term and ‘heme/oxygen binding’ under the GO_MF term (**F**). These categories were among the top 10 under the GO_BP or GO_MF terms.

GO analysis of the 1637 liver-enriched genes based on the GO_BP term (Figure 3A) showed that the top ten categories included ‘lipid metabolic process’ (53 genes, containing *mvda*, *acaca*, *lipca*, *fasn*, *cpt1ab,* etc.) (Figure 3B; Table S8), ‘negative regulation of endopeptidase activity’ (22 genes, mainly encoding peptidase inhibitors, such as *serpinf2a*, *serpina1*, *serpine1*, *serpind1*, *serping1,* etc.) (Figure 3C; Table S8), ‘blood coagulation’ (21 genes, such as *plg*, *fga*, *fgg*, *f9a*, *f10,* etc.) (Figure 3D; Table S8) and ‘complement activation’ (17 genes, mainly encoding complement components including *c3a.1*, *c3b.1*, *c4*, *c4b*, *c7b*, *c8a*, *c8b,* etc.) (Figure 3E; Table S8). Notably, 4 out of the top 10 categories were related to cholesterol/sterol biosynthesis/metabolic processes (Figure 3A). The GO_MF analysis showed that the top two categories were classified as ‘oxidoreductase activity’ (92 genes) and ‘catalytic activity’ (49 genes), respectively, which contained genes encoding enzymes involved in different metabolic reactions (Figure 3A; Table S8). Notably, ‘heme binding’ and ‘oxygen transporter activity’ were also among the top 10 categories (together 34 genes, mainly encoding hemoglobins and cytochrome p450 family members, including *hbbe1.2*, *hbbe1.3*, *hbae3*, *cyp2x7*, *cyp1a,* etc.) (Figure 3F; Table S8). The enrichment of these pathways is fully consistent with the known functions of the liver for nutrient metabolism, steroid biosynthesis and producing serum proteins [33].

We noticed that, although ‘lipid metabolic process’ under the GO_BP term and ‘oxidoreductase activity’ under the GO_MF term were among the top 10 categories in both the intestine and liver (compare Figure 2A with Figure 3A), the exact genes involved were distinct in these two organs (compare Figure 2D with Figure 3B, and Table S7 with Table S8). In addition, while the intestine expressed a variety of peptidases, the liver expressed different peptidase inhibitors (compare Figure 2C with Figure 3C). Hence, the larval intestine and liver have established their own unique transcriptomes for accomplishing their functions at 5 dpf.

### 3.3. Mining TFs Regulating the Expression of the Intestine- and Liver-Enriched Genes at 5 dpf

The above data demonstrate that the intestine tube and liver bud functionally specified at 5 dpf. TFs are context-dependent regulators of gene expression essential for cell fate specification and maintenance, and for executing cellular functions [14,34,35]. We performed a TF search (AnimalTFDB3.0) using the genes detected by our intestine and liver RNA-seq as baits and identified 263 and 208 TFs expressed in the intestine and liver, respectively, based on the cutoff criteria of an average TPM of ≧ 5, including 168 commonly expressed TFs (Tables S9 and S10). By checking the intestine TF gene list, we noticed that these TFs regulated respective cellular and biochemical pathways in the intestine. For example, *cdx1b*, *hnf4a* and *gata5* are known to regulate the development of the gut epithelium; *atoh1*, *ets2*, *her9* and family members of *klf* and *elf* are known to regulate the differentiation of absorptive and secretory intestinal epithelial cells; and *xbp1*, *smad5*, *cebpa* and *cebpb* are known to regulate the various metabolic pathways (Table S9) [2,3,14]. For the 208 liver TFs, *prox1*, *hhex, hlx* and family members of *foxa* are known to regulate the budding and differentiation of the liver; *onecut* (*hnf6*) and *hnf1b* are known to regulate the cholangiocyte differentiation; and family members of *cebp*, *ppar*, *hmgb* and *klf* are known to regulate genes involved in the hepatic metabolic pathways (Table S10) [10,36,37,38].

**Figure 4:**
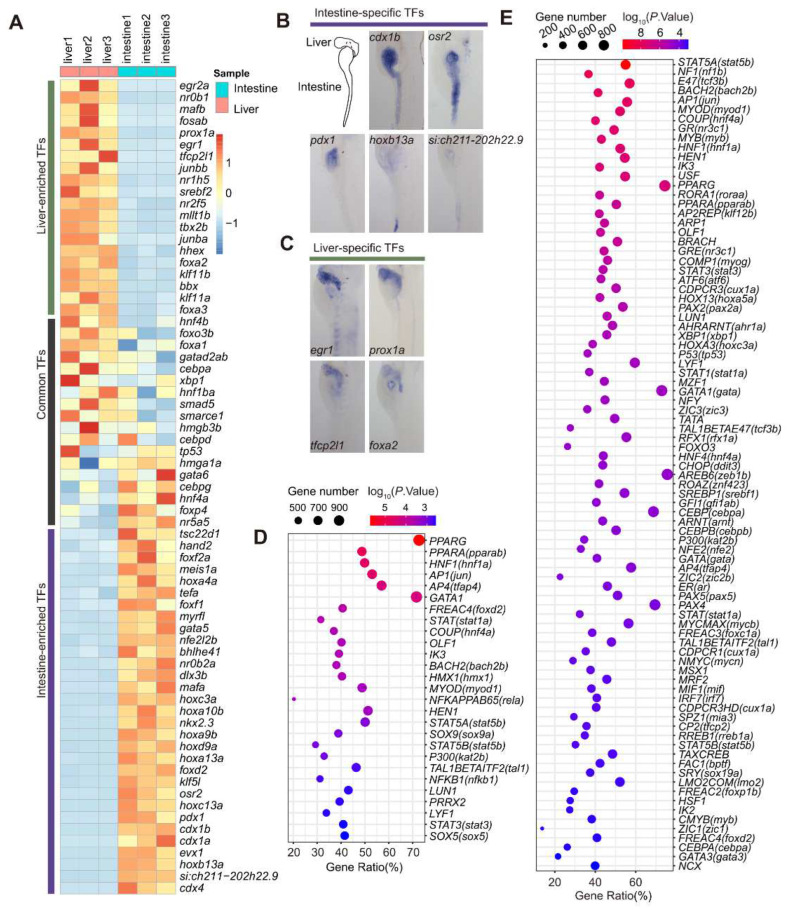
Mining TFs regulating the expression of intestine- and liver-enriched genes at 5 dpf. (**A**) Heatmap showing the hierarchical clustering of the 32 intestine-enriched, 19 liver-enriched and 18 representatives of the commonly shared TFs. (**B**,**C**) WISH showing the expression of 5 intestine-enriched and 4 liver-enriched TFs at 5 dpf. (**D**,**E**) List of putative TFs identified by TFBS analysis on David (https://david.ncifcrf.gov/tools.jsp) to regulate the expression of 2110 intestine-enriched (**D**) and 1637 liver-enriched genes (**E**). The number of genes presumably regulated by each TF and the relevant *p*-value are as indicated (*p*-value < 0.01). The names of the human TFs are provided together with a bracket showing the name of corresponding zebrafish TF (following the ENSMBL naming system).

Next, we performed another TF search within the 2110 intestine- and 1637 liver-enriched genes and identified 135 intestine-enriched and 97 liver-enriched TFs, respectively (Figure 4A, Tables S11 and S12). The 135 intestine-enriched TFs included the well-known TF genes *cdx1b* and *gata5* [3]; however, some others, including *hoxb13a*, *hoxc3a*, *osr2* and *si:ch211-202h22.9*, are yet to be characterized functionally in the intestine (Figure 4A, Table S11). Among the 97 liver-enriched TFs, *prox1a* and *hhex* are well-known liver TFs [14,36]., while *egr1*, *nr2f5* and *pparda* have not been characterized functionally in the liver (Figure 4A, Table S12). *pparda* has several paralogs, including *ppardb* in the zebrafish genome, and Pparda shares high homology with human PPARD and PPARG (Figure S2). WISH confirmed the liver-enriched expression of *pparda* in WT at 3 dpf (Figure S3A). To access the function of *pparda* in liver development, we generated a *pparda* frameshift mutant fish line via the CRISPR-Cas9 approach (Figure S3B). WISH experiments using the *fabp10a* probe revealed that only a proportion of the *pparda^zju1/zju1^* homozygous mutants displayed a mild small liver phenotype (Figure S3C,D), probably due to functional compensation by its paralogs (e.g., *ppardb*) (Figure S2). WISH using the intestinal marker *fabp2* probe and exocrine pancreas marker *trypsin* probe revealed that the mutant intestine and exocrine pancreas appeared relatively normal when compared with WT (Figure S3C). In addition to the intestine- and liver-enriched TFs, 160 TFs were found to be expressed at similar levels between the intestine and liver (|log_2_ (fold-change)| < 2), including pan-endodermal TFs, such as *foxa1*, *gata6*, *hnf4a* and *hnf4b* (Figure 4A).

To confirm the RNA-seq data, we performed a WISH experiment and demonstrated the enrichment of *cdx1b*, *osr2*, *pdx1*, *hoxb13a* and *si:ch211-202h22.9* in the intestine (Figure 4B) and *egr1*, *prox1a*, *tfcp2l1* and *foxa2* in the liver (Figure 4C). The enriched expression of *si:ch211-202h22.9* in the intestine and *egr1* and *tfcp2l1* in the liver bud at 5 dpf has not been reported previously. Interestingly, the WISH result showed that, while *cdx1b* was expressed along the entire intestine, *pdx1* was expressed in the anterior bulb and *osr2* in the posterior bulb toward the hindgut (Figure 4B). In contrast, the expression of *hoxb13a* appeared to be restricted to the proctodeum region (Figure 4B). 

Next, we submitted the 2110 intestine-enriched genes as target genes to TFBS analysis on David (https://david.ncifcrf.gov/tools.jsp) for the purpose of identifying TFs responsible for regulating the expression of these genes. This program was built based on human data, so it first needed to match the zebrafish target genes to their corresponding human orthologues and then search for their human TF regulators. In total, 1438 out of the 2110 genes had a match in the database, and 27 human TFs (*FDR* < 0.01) were identified to regulate their expression. Each TF was found to regulate multiple target genes. and each target gene could be regulated by different TFs (Figure 4D; Table S13). For example, PPARG, HNF1 and COUP regulated 1088, 747 and 554 target genes, respectively, whereas *SLC5A7* and *ACE2* can be regulated by the TFs GATA1, HNF1 and COUP (Table S13). Similarly, analysis of the 1637 liver-enriched target genes showed that 1275 qualified baits could be regulated by 126 TFs (*FDR* < 0.01) (Figure 4E; Table S14). To our surprise, a greater number of TFs were identified for the liver genes than the intestine genes, suggesting that the transcription regulation networks for liver genes might be more complicated; however, it could merely be because the datasets were more complete for the liver genes in building up the database. It was unexpected that GATA1, a key regulator of erythrocyte development, was identified to regulate the expression of the intestine-enriched genes via this analysis. This could be due to the 2110 intestine-enriched genes being obtained by comparing with the liver transcriptomes, and we could not exclude the possibility that some of these 2110 genes might have been expressed in the blood cells. Alternatively, the data used to construct the TFBS database might contain datasets obtained by GATA1 overexpression. Since GATA family members share the DNA-binding motif overexpressed GATA1, it probably activated some of the intestine genes in their samples. Nevertheless, this analysis suggests that the expression of genes in the intestine and liver is regulated by complex transcription regulatory networks [14,34,35].

### 3.4. Identification of DEGs among the Anterior, Middle and Posterior Regions of the Intestine at 5 dpf

**Figure 5:**
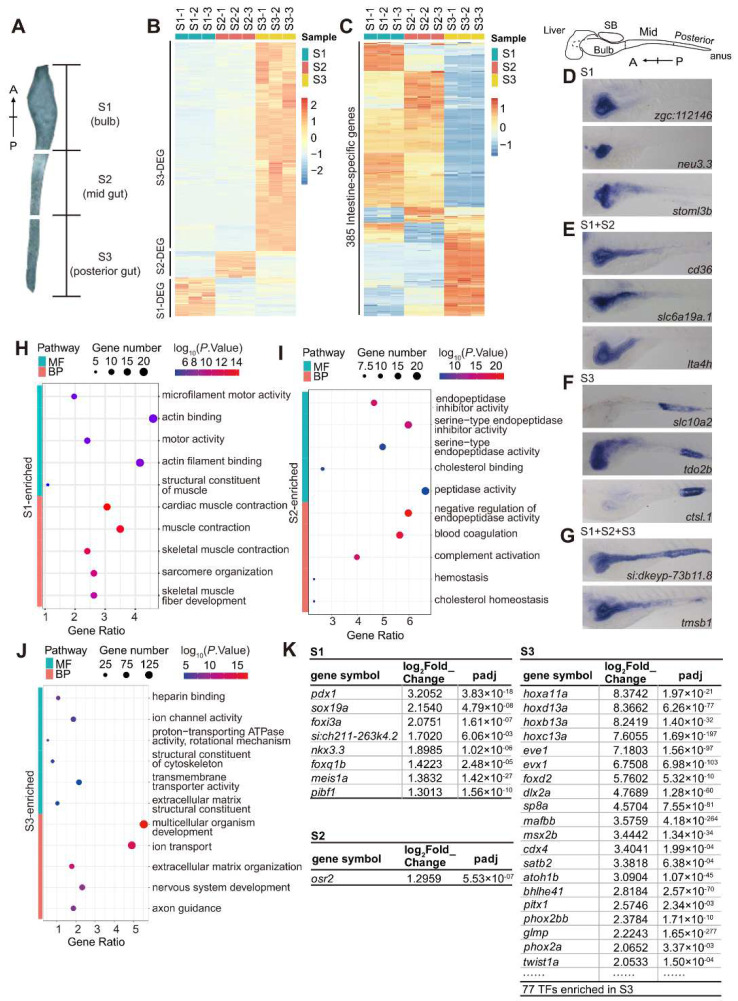
RNA-seq analysis of the gene expression profiles in the anterior (S1), middle (S2) and posterior regions (S3) of the intestine at 5 dpf. (**A**) Graph showing the region covered by the S1, S2 and S3 intestine fragments. (**B**) Hierarchical clustering heatmap comparing the 452 S1-enriched, 309 S2-enriched and 2449 S3-enriched DEGs among the three S1, S2 and S3 RNA-seq samples at 5 dpf. (**C**) Hierarchical clustering heatmap showing the distribution of 385 intestine-specific genes within the three S1, S2 and S3 RNA-seq samples at 5dpf. (**D**–**G**) WISH showing the expression of representative genes in the regions of S1 (intestinal bulb) (**D**), S1 + S2 (bulb + mid-intestine) (**E**), S3 (hindgut) (**F**) and S1+S2+S3 (entire intestinal tube) (**G**)**.** (**H**–**J**) GO analysis of the 452 S1-enriched (**H**), 309 S2-enriched (**I**) and 2449 S3-enriched (**J**) genes under the GO_BP and GO_MF terms. The top 5 categories are provided. (**K**) Information for the 8 S1-enriched, 1 S2-enriched and representatives of the 77 S3-enriched TF genes identified by RNA-seq.

To gain more insight into the gene expression profiles in different functional domains along the larval intestine, we micro-dissected the entire intestine at 5 dpf and then cut the intestine into anterior (S1), middle (S2) and posterior (S3) segments (Figure 5A). S1, S2 and S3 from 17 embryos were separately pooled and the total RNA was extracted from the pooled intestine segments for RNA-seq analysis. Analysis of the RNA-seq data from three independent samples for each segment identified, on average, 22,802, 22,997 and 23,991 expressed genes in S1, S2 and S3, respectively (Table S15, National Genomics Data Centre accession number: PRJCA006909). Unsupervised clustering analysis showed that the S1 and S2 segments displayed expression patterns more similar to each other than to that of the S3 segment (Figure S4), much like the situation in the adult intestine [20]. Enrichment analysis (|log_2_ (fold-change)| > 1 and *p* < 0.05) showed that 452 genes were enriched in S1, 309 in S2 and 2449 in S3 (Figure 5B; Tables S16–S18).

Cross-comparison of the 385 intestine-specific genes (Table S5) with the genes detected by the S1, S2 and S3 RNA-seq showed that 36 genes were notably enriched in S1 and 103 in S3. Interestingly, S1 and S2 shared a large number of the intestine-specific genes when compared with their expression in S3. Meanwhile, S1 and S2 each shared some intestine-specific genes with S3, while very few genes were detected in all three regions (Figure 5C). WISH confirmed *zgc:112146*, *neu3.3* and *stom13b* to be enriched in S1 (Figure 5D); *cd36*, *slc6a19a.1* and *lta4h* in S1 plus S2 (Figure 5E); *slc10a2*, *tdo2b* and *ctsl.1* in S3 (Figure 5F); and *si:dkeyp-73b11.8* and *tmsb1* in all three regions (Figure 5G).

GO analysis of the S1-enriched DEGs showed that the top five categories in both GO_MF and GO_BP terms were mainly related to the muscle contraction process (40 genes, including *myhb*, *myh7*, *myh7l*, *smyyc2*, *tnnc1b*, *tnni1b*, *tnnt3a,* etc.) (Figure 4H; Table S19), suggesting a strong contraction activity of the zebrafish intestinal bulb, an analog of the true stomach in higher vertebrates [19]. For the S2-enriched DEGs, GO analysis identified the peptidase activity and their inhibitors, blood coagulation, complement activation and cholesterol homeostasis to be among the top five categories in the GO_MF and GO_BP terms (Figure 5I; Table S20), suggesting that the S2 region is responsible for breaking down nutrients, such as proteins, cholesterol transportation and mucosal immunity [3,19,21]. For the S3-enriched DEGs, the top five categories in the GO_MF and GO_BP terms included the categories of ‘multicellular organism development’ (133 genes), ‘ion transport’ (118 genes) and ‘heparin binding’ (Figure 5J; Table S21), suggesting that the S3 region is still developing and carries out the function of ion absorption [3,20,21].

To identify the TFs involved in the regional specification of the intestine, we blasted the 263 intestine-expressed TFs against the 452 S1-enriched, 309 S2-enriched and 2449 S3-enriched genes. The result showed that 8, 1 and 77 TF genes were among the S1-, S2- and S3-enriched genes, respectively (Figure 5K; Table S20). Notably, *pdx1*, *sox19a* and *foxi3a* were enriched in S1; *osr2* was enriched in S2; and *cdx4*, *foxd2* and many *hoxa* and *hoxb* family members were enriched in S3 (Table S22). WISH confirmed that the expression of *pdx1* was enriched in the S1 region, *osr2* in the S2 and S3 regions and *hoxb13a* in the S3 region of the intestine at 5 dpf (Figure 4B), suggesting that these TFs are likely to be involved in functional specification or cell fate maintenance in these sub-domains.

### 3.5. scRNA-Seq Analysis Identified 10 Cell Types within the Intestine at 5 dpf

The above data clearly demonstrate that the intestinal cells in the S1, S2 and S3 regions exhibit distinctive molecular features. Previous reports have shown that the adult zebrafish intestine inner epithelium is composed of absorptive enterocytes, secretory goblet cells, enteroendocrine cells and stem cells, surrounded by smooth muscle cells, enteric nerves, resident immune cells, etc. [3,19,21,39,40]. To better understand the relationship between the intestinal cells in different functional domains and to identify more feature genes to mark different cell types in the zebrafish larvae intestine, we micro-dissected entire intestines from WT at 5 dpf and performed an scRNA-seq analysis (customized microwell-seq method) [31]. A total of 1229 cells met the quality control criteria (minimum of 200 genes and less than 20% mitochondrial genes per cell) and were selected for data analysis (Figure S5) (National Genomics Data Centre accession number: PRJCA006909).

**Figure 6:**
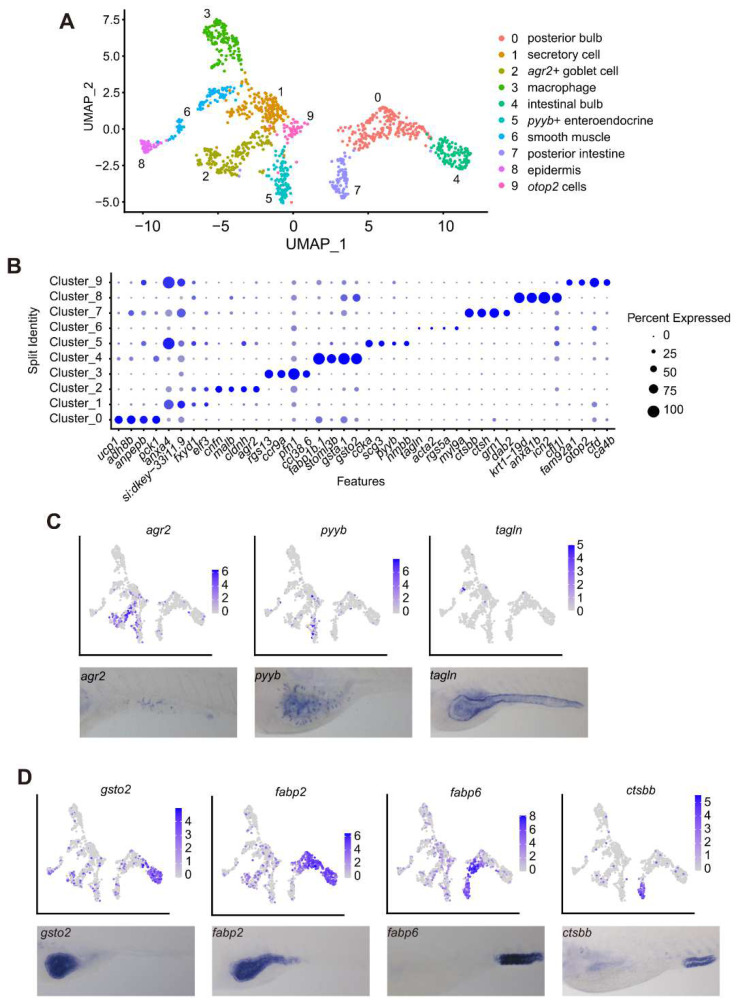
scRNA-seq analysis identified 10 cell types in the WT intestine at 5 dpf. (**A**) Visualization of integrated unsupervised clustering of cells in the UMAP plot of the sequenced 1229 cells from the WT intestines (**left**), together with cell annotations (**right**). (**B**) Dot plot of 4 marker genes for each cell cluster (cluster 0–cluster 9) for cell type annotation. (**C**,**D**) Feature plot together with WISH showing the expression of feature genes in the goblet (*agr2*), enteroendocrine (*pyyb*) and smooth muscle cells (*tagln*) (**C**), and in the intestinal bulb (*gsto2*), posterior bulb plus mid-intestine (*fabp2*, *fabp6*) and hindgut (*ctsbb*) cells (**D**).

UMAP of the scRNA-seq data of the 1229 cells grouped the cells into 10 clusters (cluster 0–9) (Figure 6A, left). The dot plot based on the feature genes (Figure S6, Table S23) showed that these 10 cell clusters represented 10 cell types, including goblet cells (cluster 2), immune cells (cluster 3), enteroendocrine cells (cluster 5), smooth muscle cells (cluster 6), epidermal cells (cluster 8) and newly identified Otop2 cells [21] (cluster 9) (Figure 6A,B). WISH using the feature genes as probes defined the distributions of the goblet cells (*agr2^+^*), enteroendocrine cells (*pyyb^+^*) and smooth muscle cells (*tagln^+^*), respectively, along the intestine (Figure 6C).

Clusters 0, 4 and 7 represented functionally differentiated absorptive enterocytes (Figure 6A,B; Figure S6). WISH using the cluster 4 feature gene *gsto2* revealed its higher expression in the intestinal bulb (Figure 6D). In contrast, the expression of cluster 7 feature genes *ctsbb* was more restricted in the hindgut (Figure 6D). *dab2* was also a feature gene for the cluster 7 cells (Figure 6B), suggesting that cluster 7 contained the recently defined lysosome-rich enterocytes (LRE) [19,21]. Interestingly, cluster 0 cells were not uniform in their gene features; for example, some feature genes (*ucp1*, *cyp7a1*) appeared to be restricted in the cluster 0 cells, while some others were shared with cluster 4 (*apoc2*, *apoa4b.1*, *chia.2* and *fabp2,* etc.) or with cluster 7 (*fabp6*, *grn1* and *epd12,* etc.) (Figure S6). Based on the expression patterns of *fabp2* and *fabp6* (Figure 6D), we suggest that the cluster 0 cells might be from the mid-intestine, which can execute broad cellular functions.

The cluster 1 feature genes were shared by the goblet, enteroendocrine, hindgut and Best4/Otop2 cells (Figure 6B; Figure S6). Considering the fact that the intestine tissue was harvested at 5 dpf, when the intestine was still developing, cluster 1 cells might represent a group of fate-uncommitted multi-functional cells that might have the potential to be differentiated further into a more specific cell type later, or these cells might be temporal at this development stage and might be replaced by new, more specifically differentiated cells during the process of intestine maturation. This is worth further investigation in the future. Our scRNA-seq data identified characteristic molecular features to distinguish the different epithelial cells along the intestinal tube at 5 dpf. 

### 3.6. scRNA-seq Analysis Identified Eight Cell Types within the Liver bud at 5 dpf

**Figure 7:**
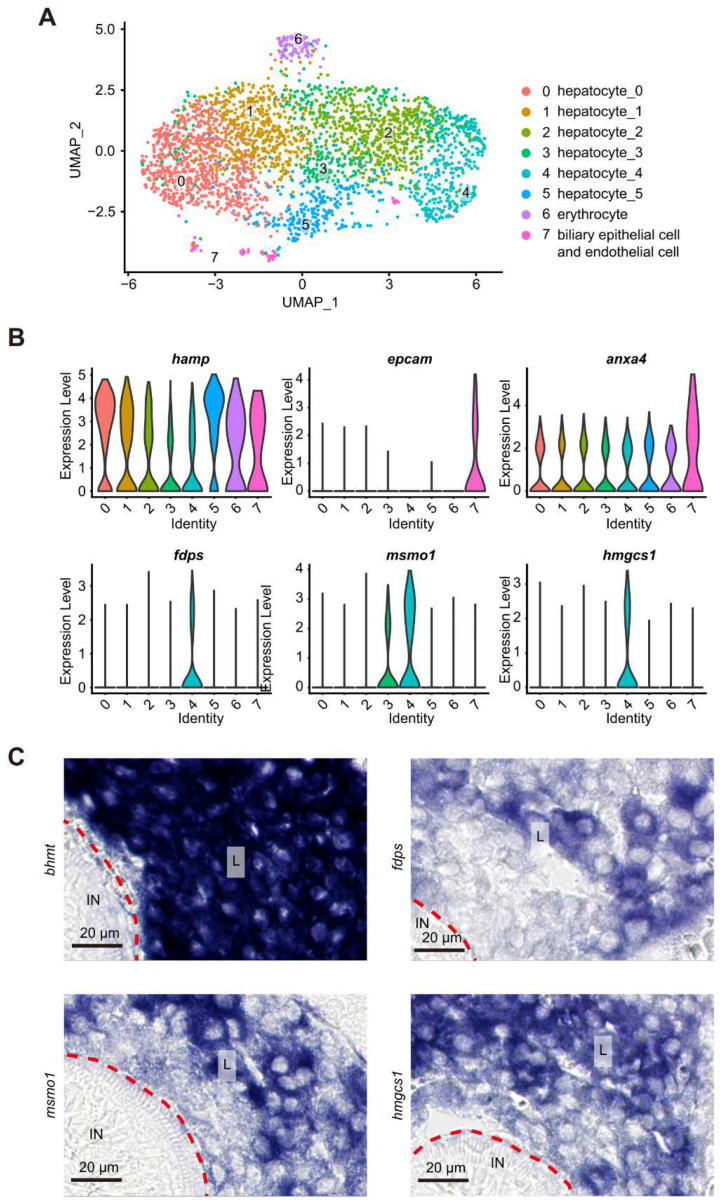
scRNA-seq analysis identified 8 cell clusters in the WT liver at 5 dpf. (**A**) Visualization of integrated unsupervised clustering of cells in the UMAP plot of the sequenced 3045 cells from the WT liver. (**B**) Violin graph showing the expression of *hamp* in all cell types, of *faps* and *hmgcs1* in cluster 4, of *msmo1* in both cluster 3 and 4 cells and of *epcam* in cluster 7 (cholangiocytes). (**C**) Sections of embryos after WISH using the probes for detecting the transcripts of *bhmt* (pan-hepatocyte marker), *faps*, *hmgcs1* and *msmo1*. Note that *bhmt* was expressed abundantly in the liver bud, whereas *faps*, *hmgcs1* and *msmo1* were expressed only in a proportion of cells in the liver bud. A red dashed line separates the liver region (L) and intestinal lumen (IN).

Currently, there is no specific cell atlas for the zebrafish liver bud at 5 dpf [21,41]. The liver bud was micro-dissected from WT larvae at 5 dpf and subjected to scRNA-seq analysis [31,32]. A total of 3045 cells met the quality control criteria (minimum of 300 genes and less than 20% mitochondrial genes per cell) and were selected for data analysis (Figure S7) (National Genomics Data Centre accession number: PRJCA006909). Analyzing the scRNA-seq data based on gene features (Figure S8, Table S22) identified eight cell types (clusters 0 to 7) (Figure 7A). To our surprise, the annotation of cell clusters with feature genes identified six clusters (0 to 5) belonging to the hepatocytes (Figure 7A). Based on the heatmap of feature genes, it appeared that clusters 0 and 1 were close to each other, as were clusters 2 and 3 (Figure S8). Cluster 4 appeared to be related to clusters 2 and 3, but had unique genes. For example, the violin plot showed that *fdps* and *hmgcs1* were highly enriched in the cluster 4 hepatocytes (Figure 7B), whereas *msmo1* was enriched in both cluster 3 and 4 hepatocytes (Figure 7B). The sectioning of the WISH embryos showed that, while *bhmt* was ubiquitously expressed in all hepatocytes, *fdps*, *hmgcs1* and *msmo1* were expressed only in a proportion of the hepatocytes (Figure 7C). The remaining two clusters represented erythrocytes (cluster 6) and cholangiocytes (cluster 7) (Figure 7A). Notably, in addition to *epcam* (Figure 7B), we identified *CR318588.4* and *pfn1* to be the molecular markers for the larval cholangiocytes (Figure S8).

### 3.7. Cellular Expression Specificities of the 385 Intestine-Specific and 308 Liver-Specific Genes

**Figure 8:**
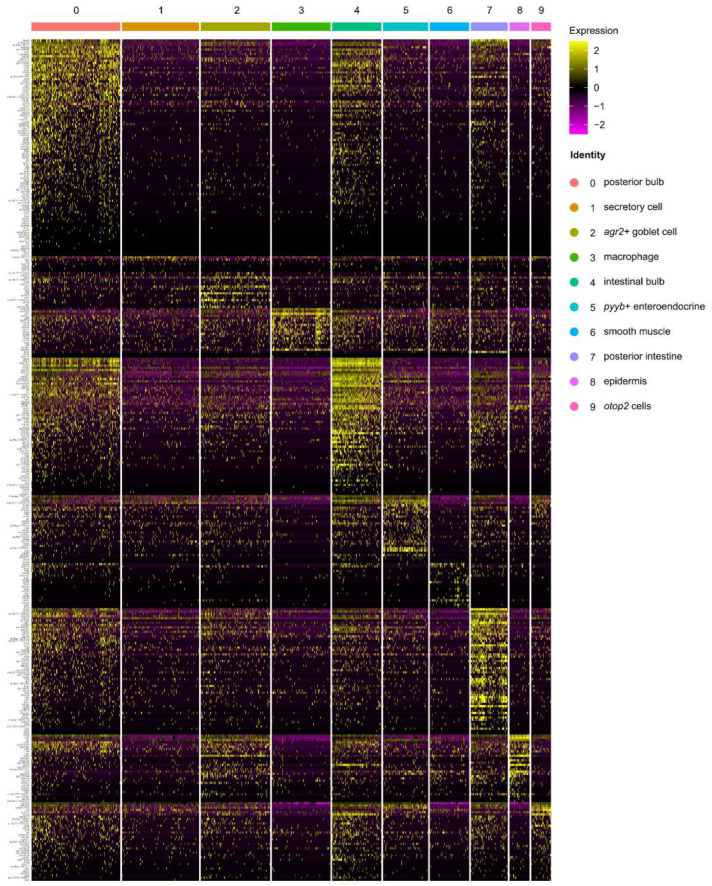
Cellular expression specificities of the 385 intestine-specific genes. Heatmap showing the expression of the 375 intestine-specific genes (10 genes were not detected by the intestine scRNA-seq) at the single-cell level in each cell cluster identified by the intestine scRNA-seq.

To identify more feature genes for each cell type in the intestine and liver at 5 dpf, we examined the expression of the 385 intestine-specific and 308 liver-specific genes in each individual cell acquired by the intestine and liver scRNA-seq, respectively. The scenario was that these genes were obtained from RNA-seq analysis by applying stringent criteria for higher specificity (|log_2_ (fold-change)| > 2) and more abundancy (TPM > 50) between the intestine and liver (Tables S5 and S6). We first examined the expression of the 385 intestine-specific genes. Overall, 10 genes were not detected by the intestine scRNA-seq; the remaining 375 genes were differentially expressed in different cell clusters, except for cluster 1 (Figure 8), further suggesting that cluster 1 might be an undefined cell type at the larvae stage (Figure 6A,B). Notably, although different in terms of abundance, the majority of the 375 intestine-enriched genes were obviously enriched in the absorptive cells represented by clusters 0 (mid-intestine), 4 (intestinal bulb) and 7 (hindgut). Meanwhile, fully consistent with the RNA-seq analysis of the S1, S2 and S3 segments, it appeared that cluster 0 and cluster 4 cells displayed similar expression patterns for the 375 intestine-specific genes, and cluster 0 and cluster 7 cells shared a fraction of these genes (Figure 8). Detailed analysis revealed that the feature genes of cluster 0 (*adh8b*, *anpepb* and *pck1*), cluster 2 (goblet cells, *icn*, *cnfn* and *agr2*), cluster 3 (immune cells, *pfn1*), cluster 4 (*fabp1b.1*, *stoml3b* and *gsta.1*), cluster 5 (enteroendocrine cells, *pyyb*, *insl5a* and *ccka*), cluster 6 (smooth muscle, *tagln*, *acta2* and *myl9a*), cluster 7 (*ctsbb*, *ctsh* and *dab2*), cluster 8 (epidermis, *cfl1l* and *gstp2*) and cluster 9 (Otop2 cells, *cfd* and *ca4b*) were among the 385 intestine-specific genes (compare Figure 6B with Figure 8). Therefore, the analysis not only validated the cell types for the intestine at 5 dpf, but also assigned the cellular specificity of the 375 intestine-specific genes.

Next, we examined the expression of 308 liver-specific genes at the single-cell level (Figure S9). Thirteen genes were not detected by the liver scRNA-seq. Surprisingly, except for the erythrocytes (cluster 6), the remaining 295 genes lacked an obvious pattern of enrichment in the rest cell clusters (Figure S9), suggesting that, although our scRNA-seq separated the hepatocytes into six subtypes, these hepatocytes expressed a great range of similar functional genes (Figure S9).

## 4. Discussion

By 5 dpf, the zebrafish digestive system has been well established to accomplish its physiological function and meanwhile continues their development. In this report, we obtained the gene expression profiles in the zebrafish intestine and liver bud at 5 dpf. Our data are complementary to previous reports [3,20,21,41], which allowed us to not only understand the genes required for executing the physiological function, but also genes for encoding signaling molecules and TFs for continuous development and for determining the specific function in these two organs at 5 dpf. Data analysis showed that the liver bud and intestine not only shared a large number of house-keeping genes for maintaining routine cellular activities, but also some common endoderm identity genes, including pan-endodermal TFs, such as Gata factors (Gata4/5/6) and FoxA factors (FoxA1/2/3), which are essential for the specification and development of the entire zebrafish digestive system [1,16,33,42,43,44]. Data analysis also clearly showed that the intestine and liver bud at 5 dpf expressed the intestine- or liver-enriched genes related to signal transduction. For example, among the intestine-enriched genes, *efna2a* and *CT990561.1* related to Ephrin signaling, which is essential for the establishment of the intestinal lumen, were identified [17]. Among the liver-enriched genes, *bmpr2b* related to Bmp signaling, *fgf10b* related to Fgf signaling and *dkk3a* related to Wnt signaling, which are known to be essential for liver development and function, were identified [9,45,46,47]. *Notch1b* and *jag1a,* related to Notch signaling during the development of the biliary system, were also identified among the liver-enriched genes [48,49]. The larval intestine and liver also adopted distinctive categories of TFs for their fate specification. For example, among the liver-enriched genes, *hhex* and *prox1a*, two well-known genes for liver development, as well as *stat5a,* were identified [13,14,50]. On the other hand, *pdx1* and *cdx1b,* for intestinal development and functioning, were identified among the intestine-enriched genes [14,51,52,53].

Based on morphology and molecular markers, previous studies have divided the zebrafish intestine into the intestinal bulb, mid-intestine, hindgut and proctodeum regions [5,19]. A recent report based on scRNA-seq data derived from a mixture of the liver and intestine dissected from zebrafish larvae at 6pf identified 18 cell types, including enterocytes, goblet cells and enteroendocrine cells, the three main cell types in the gut epithelia [21]. They further divided the enterocytes into five sub-groups, but without determining their corresponding positions along the intestine. Our scRNA-seq clearly identified the clusters that appeared to match the sub-regions in the intestine [5]. Our WISH using gene-specific probes determined the positions of these cell clusters along the intestine. For example, *neu3.3*- and *pdx1*-positive cells were located at the anterior region of the intestinal bulb. Because the hepatopancreatic duct was fused with the intestine at the anterior region of the intestinal bulb, these cells might function to collect the secreted products from the liver and pancreas [14,19]. Plotting the 385 intestine-specific genes against each individual cell together with the WISH experiments, we identified that *tmsb1*-positive cells were located at the posterior region of the intestinal bulb, while *osr2*-positive cells were at the mid-intestine region. The hindgut was marked by *slc10a2*-positive cells, while the proctodeum was marked by *stk25a*- and *ctsl.1*-positive cells. Based on this new knowledge, future studies will focus on how these cells are specialized, especially regarding the genetic and signaling network formed by specific signaling molecules and TFs.

## Data Availability

The data presented in this study are available on request from the corresponding author. The RNA-seq and ATAC-seq data have been deposited in the National Genomics Data Centre (https://ngdc.cncb.ac.cn/browse) with one unique accession number (PRJCA006909).

## Supplementary Materials

The following supporting information can be downloaded at: https://www.mdpi.com/article/10.3390/cells11203290/s1. Figure S1: Heatmap showing the hierarchical clustering of all genes detected in three intestine and three liver RNA-seq samples; Figure S2: Alignment of the amino acid sequences of zebrafish (Zf) Pparda, Ppardb, Pparaa and Pparab, and human (Hu) PPARD and PPARG. The corresponding gene accession number is provided at the end. Identical amino acids are highlighted with a ‘*’ in the row of ClustalCo. Figure S3: Loss-of-function of *pparda* leads to a small liver phenotype. (A) WISH showing the liver-enriched expression of *pparda* in WT at 3 dpf. (B) Diagrams showing the *pparda* genomic structure on Chr 22 (top), the gRNA target sequence (underlined in WT sequence) and 7 bp deletion in the *pparda^zju1^* mutant (middle) and WT Pparda and the resultant mutant peptide (bottom). TGA in red: premature stop codon in the *pparda^zju1^* mutant. (C) Representative WISH image comparing the development of liver (*fabp10a* probe), intestine (*fabp2* probe) and exocrine pancreas (*trypsin* probe) between WT and *pparda^zju1^*. The number of larvae showing the WISH pattern out of total larvae examined is provided in the bottom right corner. (D) Statistical analysis of the *fabp10a* signal in WT and *pparda^zju1^*. ****unpaired *t*-test *p* < 0.0001. Figure S4: Heatmap showing the hierarchical clustering of the TPM-based top 10,000 genes detected in the S1, S2 and S3 RNA-seq samples. Figure S5: Quality analysis of the scRNA-seq data from the WT intestines at 5 dpf. The number of identified unique genes (top left), number of UMI transcripts (top right) and mitochondrial gene percentage (bottom left) in each cell are plotted. The corresponding cell types annotated are shown in the bottom right. Figure S6: Heatmap showing the top differentially expressed genes in each cell cluster identified in the WT intestines by scRNA-seq. Transcriptomes from 1229 WT intestine cells were used to construct the heatmap using the Seurat package. Figure S7. Quality analysis of the scRNA-seq data from the WT liver at 5 dpf. The number of identified unique genes (left), number of UMI transcripts (middle panel) and mitochondrial gene percentage (right) in each cell are plotted. Corresponding cell types annotated are shown in the far right. Figure S8. Heatmap showing the top differentially expressed genes in each cell cluster identified in the WT liver by scRNA-seq. Transcriptomes from 3045 WT liver cells were used to construct the heatmap using the Seurat package. Figure S9. Cellular expression specificities of the 308 liver-specific genes. Heatmap showing the expression of 308 liver-specific genes at the single-cell level in each cell cluster identified by the liver scRNA-seq. Table S1: List of primer sequences for cloning gene probes for WISH. Table S2: Quality analysis of the RNA-seq data and mapping information for the clean reads for the intestine and liver samples. Table S3: List of the 2110 intestine-enriched genes. Table S4: List of the 1637 liver-enriched genes. Table S5: List of the 385 intestine-specific genes identified at 5 dpf. Table S6: List of the 308 liver-specific genes identified at 5 dpf. Table S7: Information for the top 10 categories under the GO_MF and GO_BP terms for the 2110 intestine-enriched genes. Table S8: Information for the top 10 categories under the GO_MF and GO_BP terms for the 1637 liver-enriched genes. Table S9: List of the 263 TF genes expressed in the intestine identified at 5 dpf. Table S10: List of the 208 TF genes expressed in the liver identified at 5 dpf. Table S11: List of the 135 intestine-enriched TF genes identified at 5 dpf. Table S12: List of the 97 liver-enriched TF genes identified at 5 dpf. Table S13: List of TFs regulating the expression of the 2110 intestine-enriched genes. Table S14: List of TFs regulating the expression of the 1637 liver-enriched genes. Table S15: Quality analysis of the RNA-seq data and mapping information for the clean reads of the S1, S2 and S3 segments. Table S16: List of the 452 S1-enriched genes identified at 5 dpf. Table S17: List of the 309 S2-enriched genes identified at 5 dpf. Table S18: List of the 2449 S3-enriched genes identified at 5 dpf. Table S19: Information for the top 5 categories under the GO_BP and GO_MF terms for the 452 S1-enriched genes. Table S20: Information for the top 5 categories under the GO_BP and GO_MF terms for the 309 S2-enriched genes. Table S21: Information for the top 5 categories under the GO_BP and GO_MF terms for the 2449 S3-enriched genes. Table S22: List of S1-, S2- and S3-enriched TFs identified at 5 dpf. Table S23: List of feature genes for the 10 cell clusters identified by the intestine scRNA-seq. Table S24: List of feature genes for the 8 cell clusters identified by the liver scRNA-seq.
