# Supplementary figures and images for: Unraveling Differential Transcriptomes and Cell Types in Zebrafish Larvae Intestine and Liver
Source: Cells. 2022 Oct 19;11(20):3290. doi: 10.3390/cells11203290 (PMC9600436; doi:10.3390/cells11203290)

Figure S1

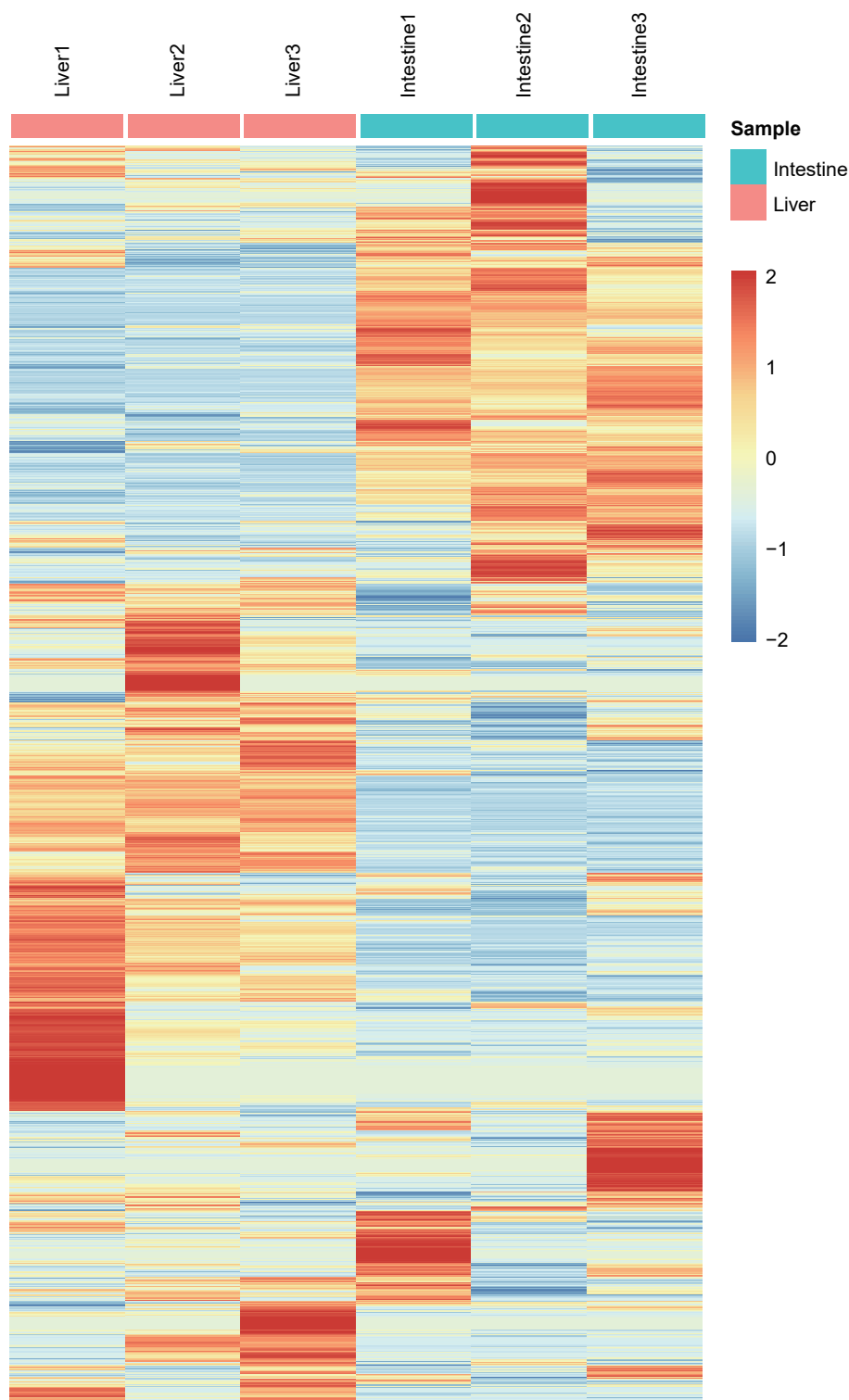

[illegible]

Figure S3

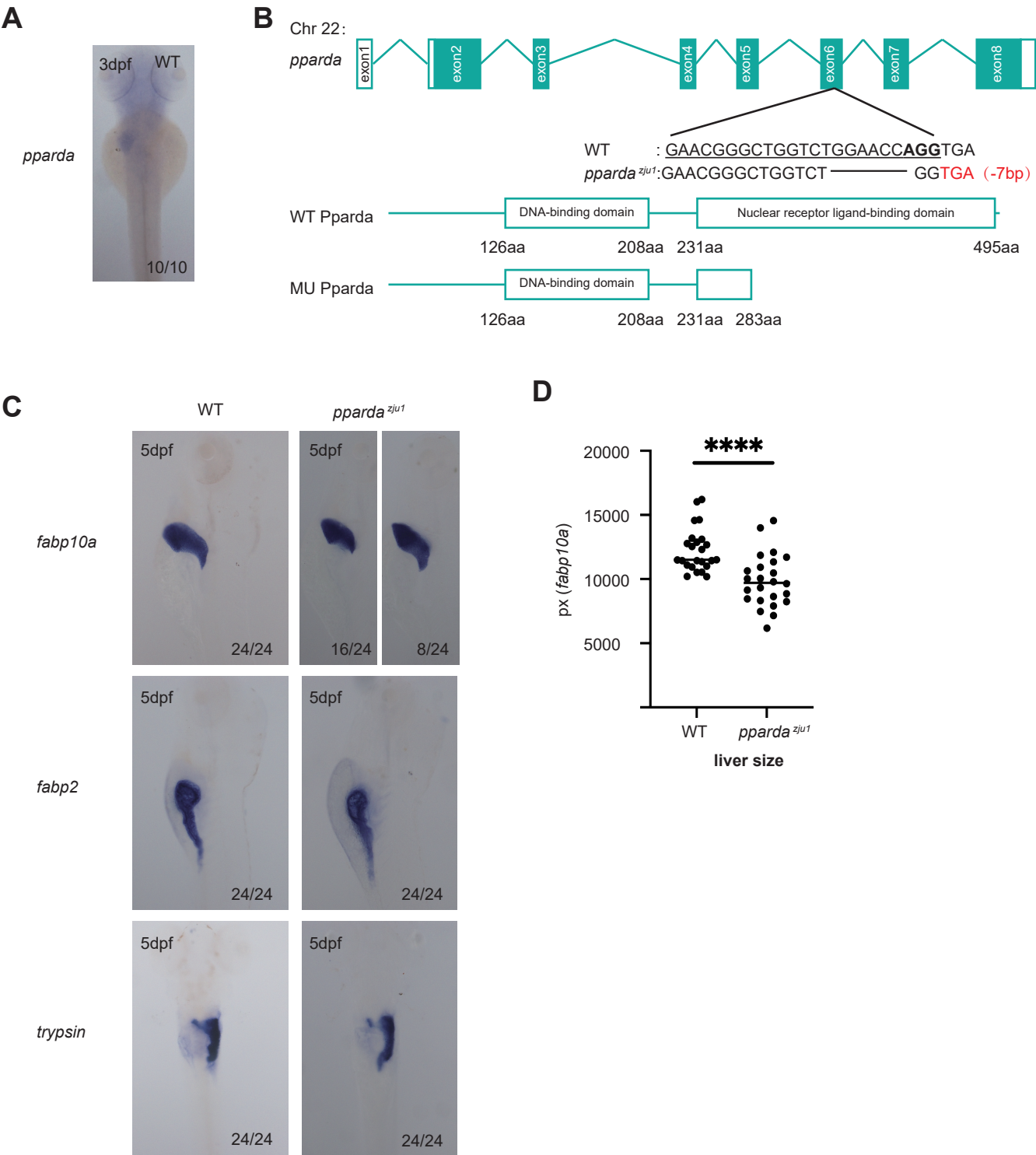

Figure S4

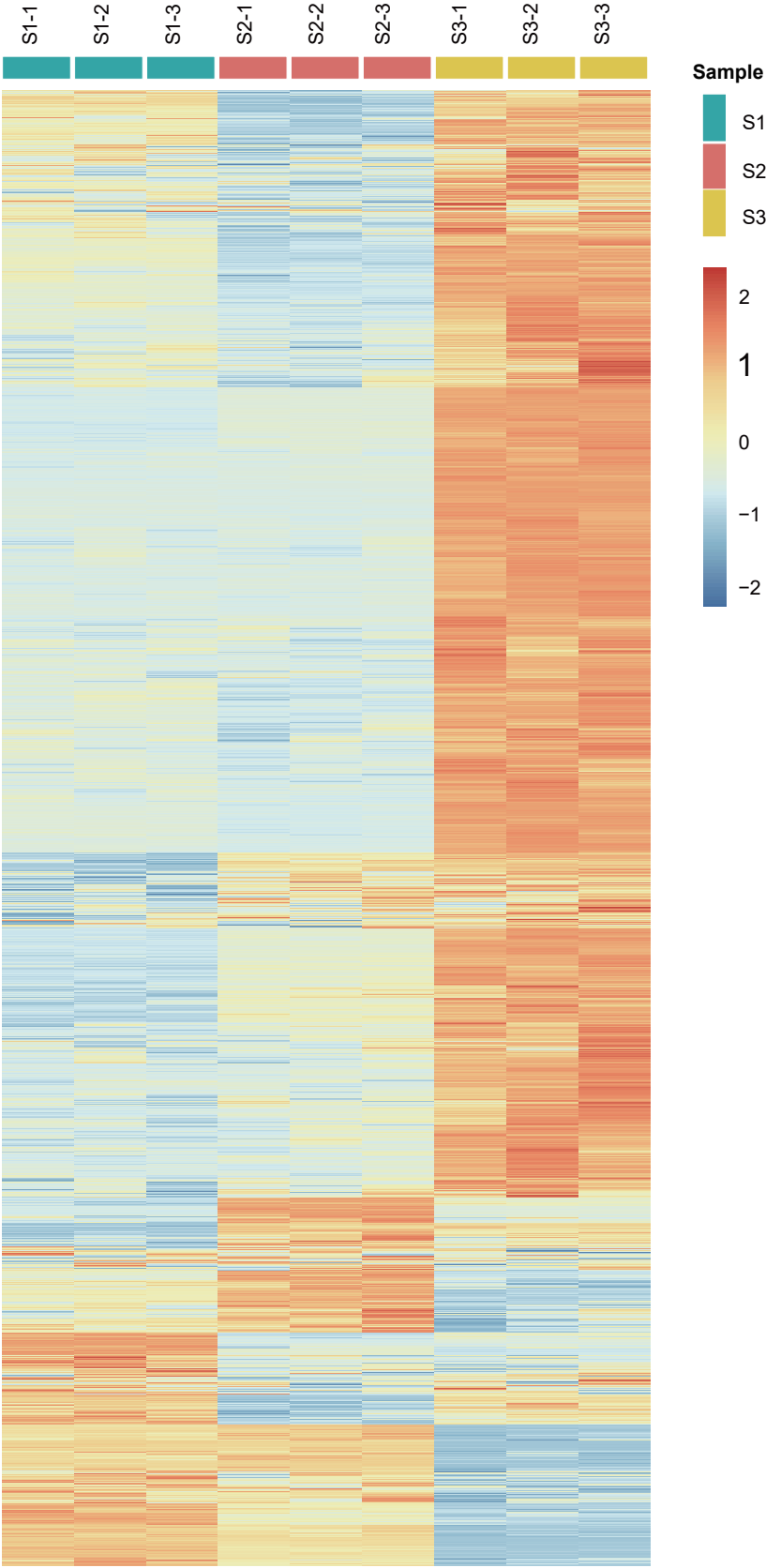

Figure S5

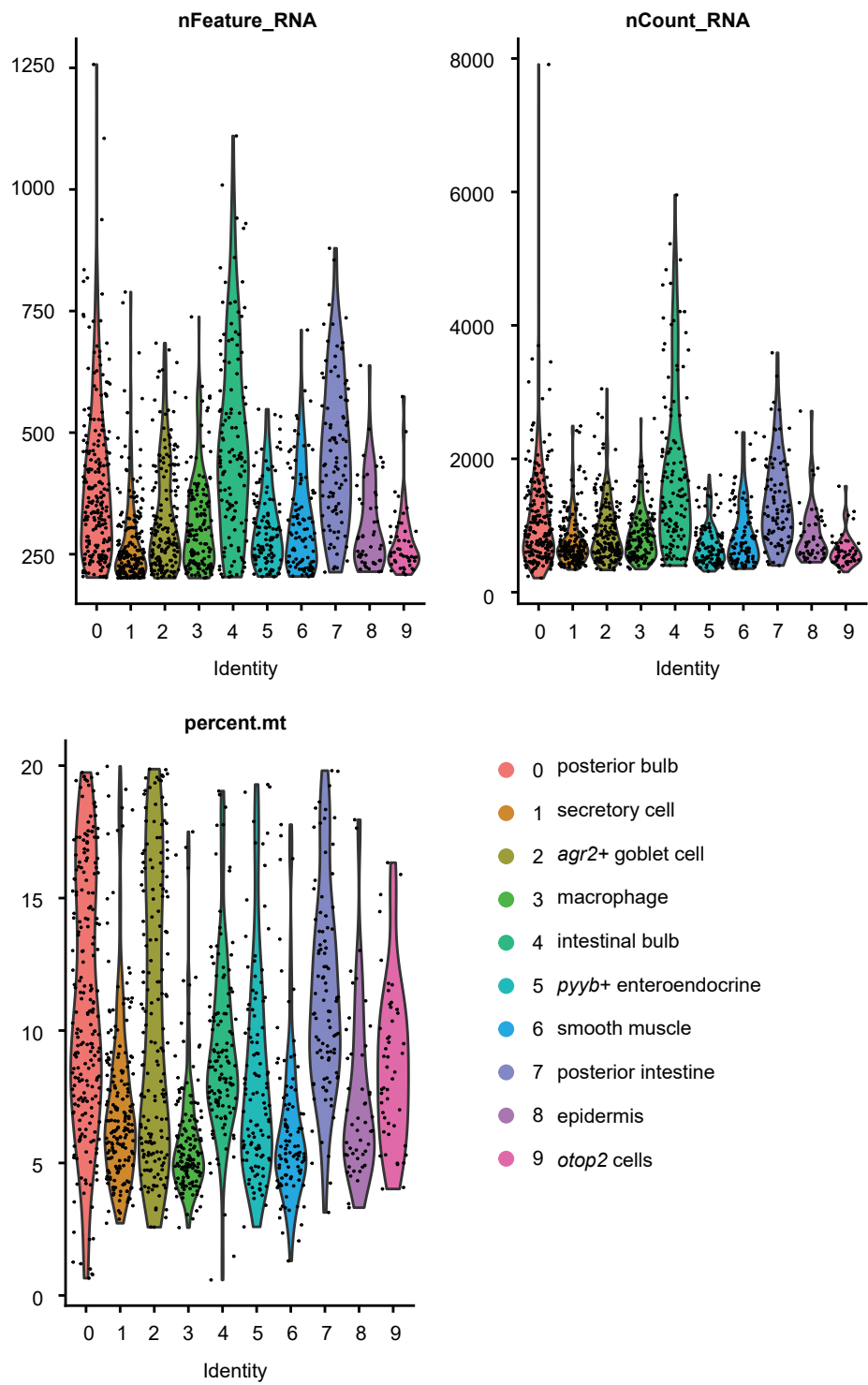

Figure S6

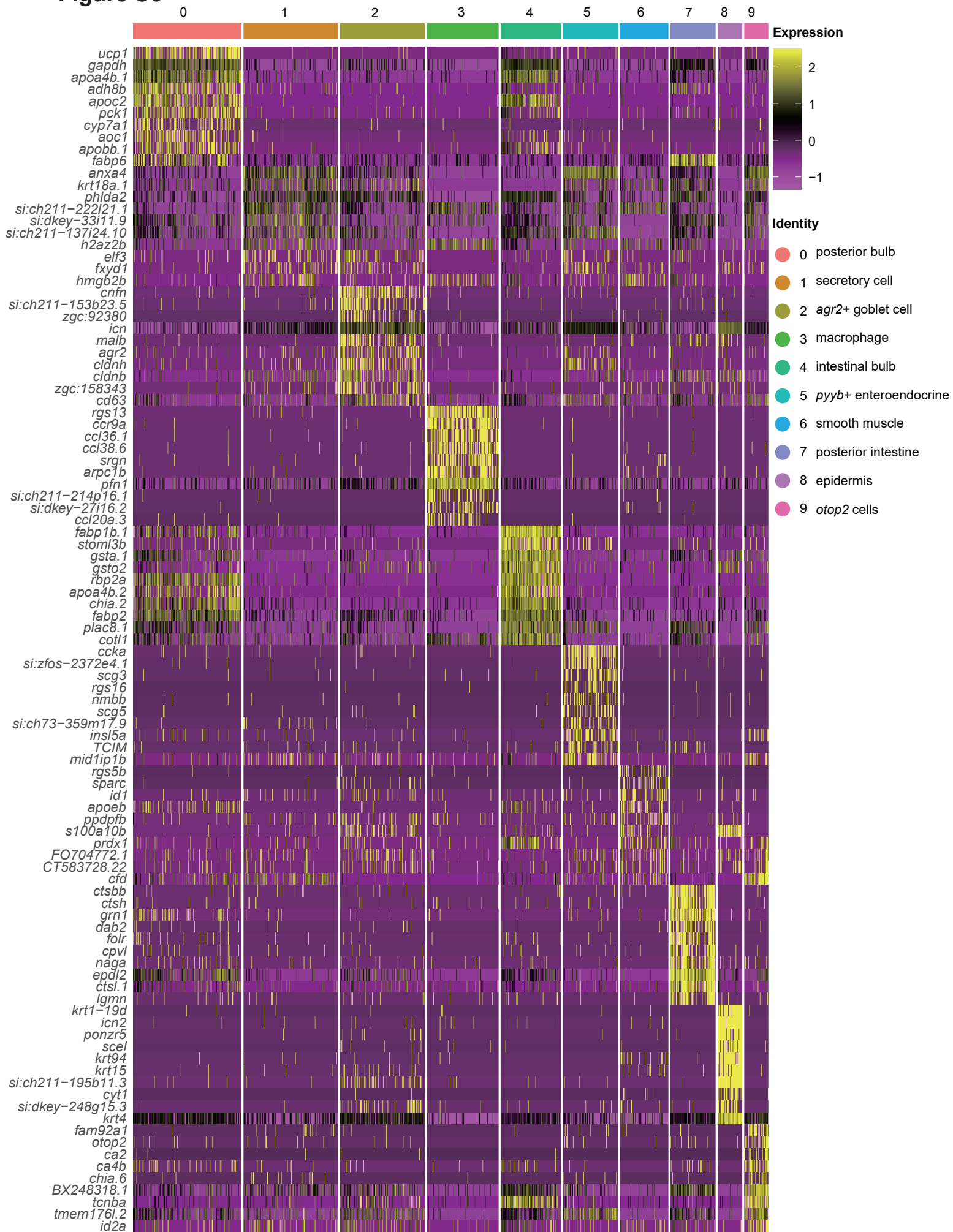

Figure S7

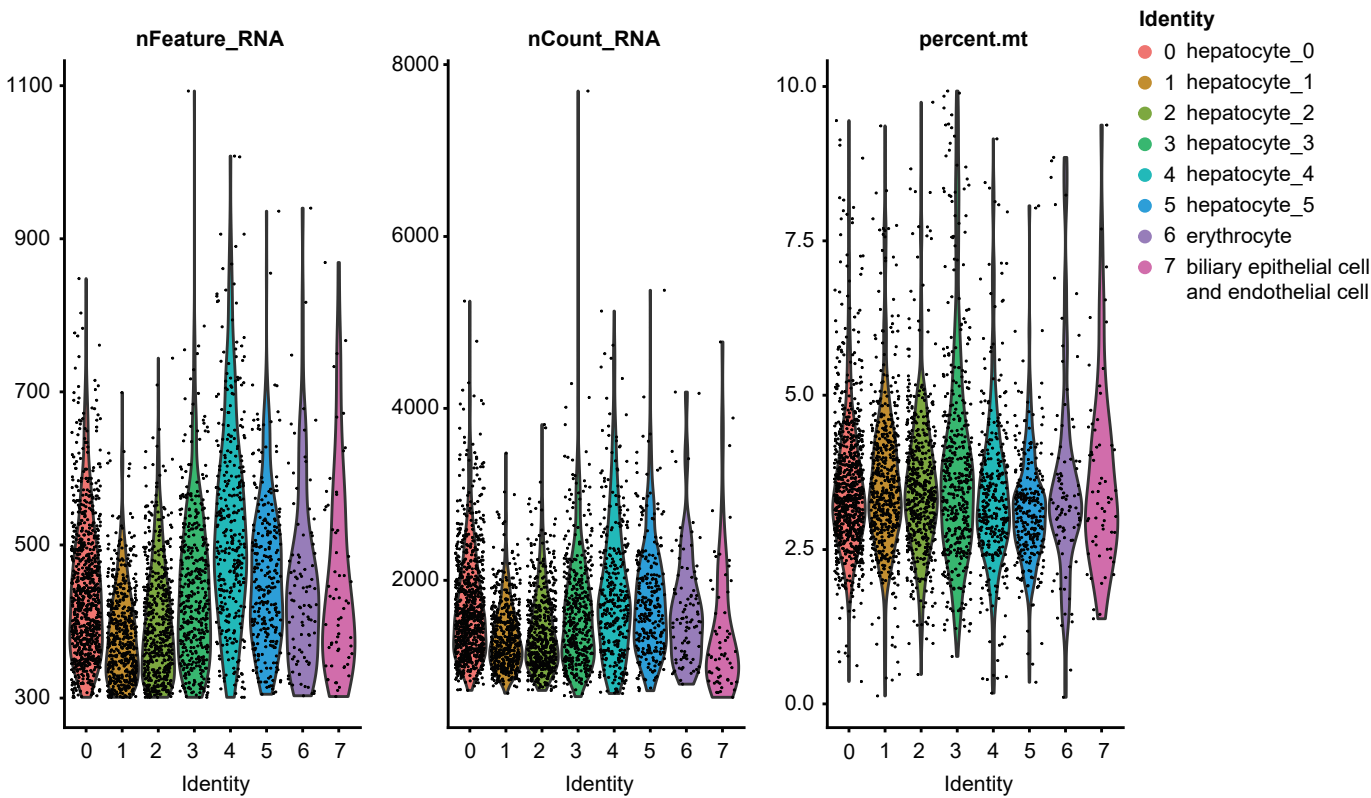

Figure S8

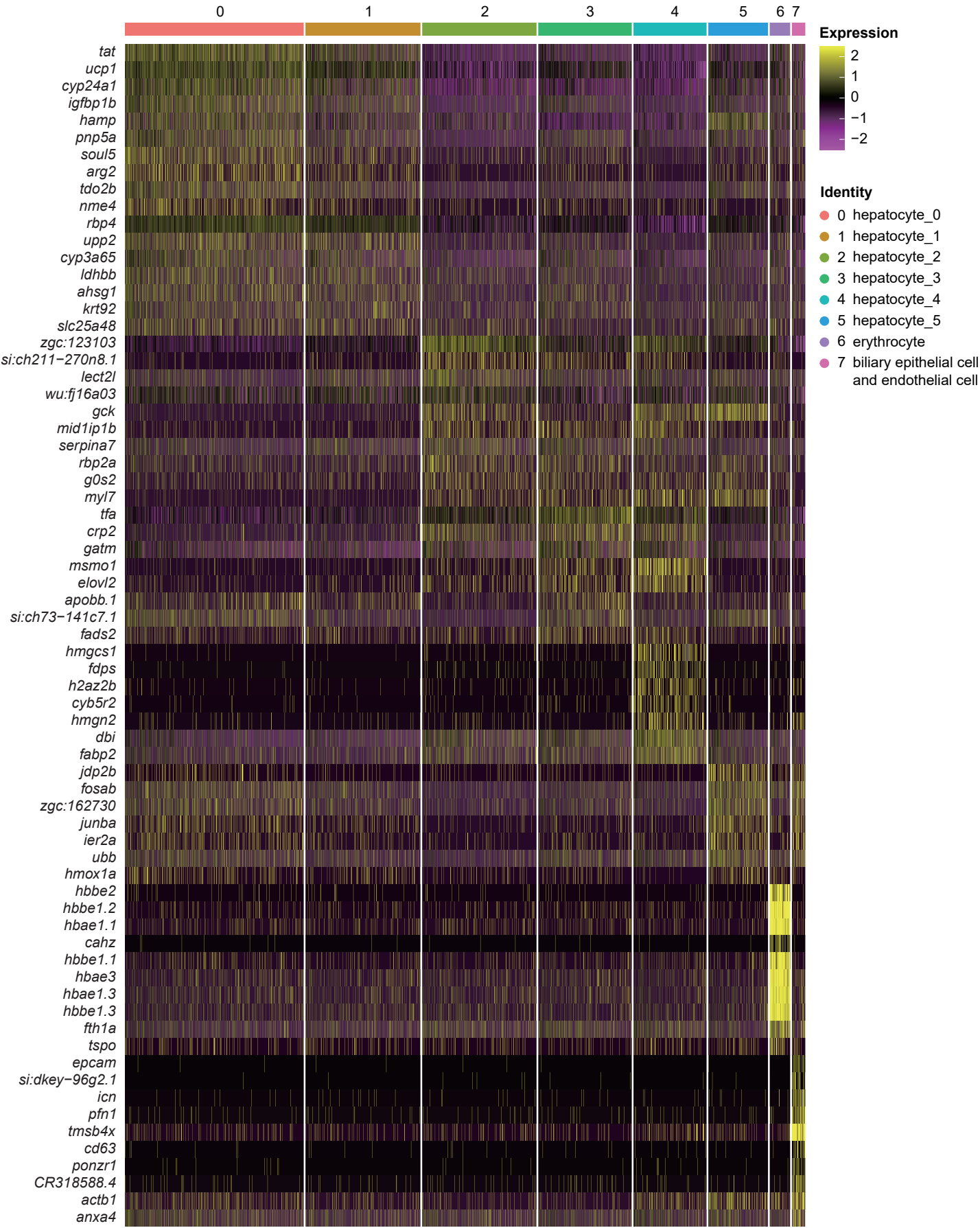

### Figure S9

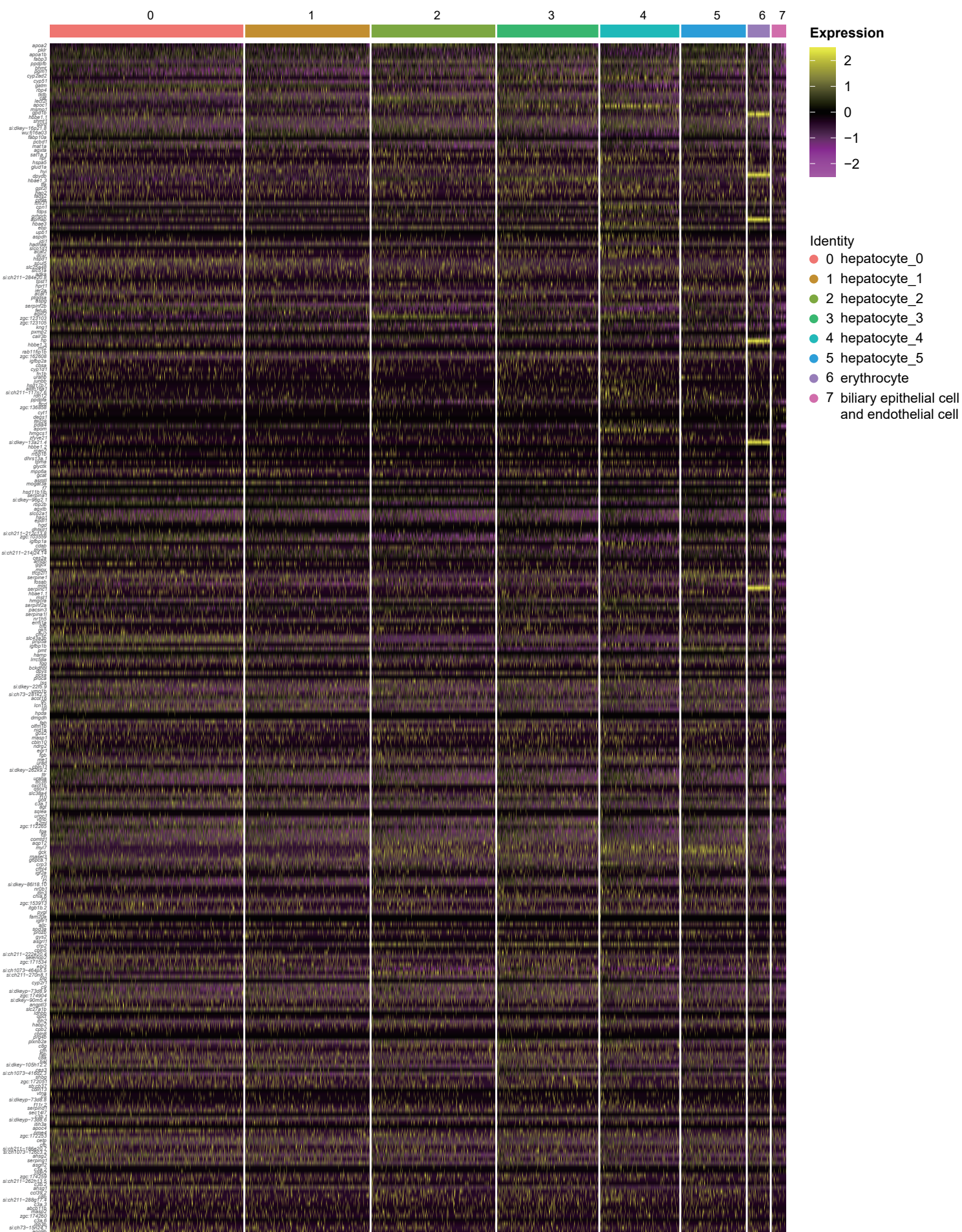

Supplement: Supplementary file 1 [file cells-11-03290-s001.zip › Figures S1-S9.pdf]
